# Circulating Tumor DNA-Based Genomic Profiling Assays in Adult Solid Tumors for Precision Oncology: Recent Advancements and Future Challenges

**DOI:** 10.3390/cancers14133275

**Published:** 2022-07-04

**Authors:** Hiu Ting Chan, Yoon Ming Chin, Siew-Kee Low

**Affiliations:** 1 Project for Development of Liquid Biopsy Diagnosis, Cancer Precision Medicine Center, Japanese Foundation for Cancer Research, Tokyo 135-8550, Japan; yoonming.chin@jfcr.or.jp (Y.M.C.); siewkee.low@jfcr.or.jp (S.-K.L.); 2 Cancer Precision Medicine, Inc., Kawasaki 213-0012, Japan

**Keywords:** circulating tumor DNA, liquid biopsy, actionable alterations, genomic biomarkers

## Abstract

**Simple Summary:**

The use of liquid biopsy for tumor genomic profiling to identify genomic biomarkers for targeted therapies has revolutionized the clinical practice in oncology management. In this review, we have summarized the recent advancements of liquid biopsy-based genomic profiling that have led to their approval for treatment selection in advanced cancer patients and highlighted the major factors that should be considered to choose the most appropriate genomic profiling assay for different patients under different clinical conditions.

**Abstract:**

Genomic profiling using tumor biopsies remains the standard approach for the selection of approved molecular targeted therapies. However, this is often limited by its invasiveness, feasibility, and poor sample quality. Liquid biopsies provide a less invasive approach while capturing a contemporaneous and comprehensive tumor genomic profile. Recent advancements in the detection of circulating tumor DNA (ctDNA) from plasma samples at satisfactory sensitivity, specificity, and detection concordance to tumor tissues have facilitated the approval of ctDNA-based genomic profiling to be integrated into regular clinical practice. The recent approval of both single-gene and multigene assays to detect genetic biomarkers from plasma cell-free DNA (cfDNA) as companion diagnostic tools for molecular targeted therapies has transformed the therapeutic decision-making procedure for advanced solid tumors. Despite the increasing use of cfDNA-based molecular profiling, there is an ongoing debate about a ‘plasma first’ or ‘tissue first’ approach toward genomic testing for advanced solid malignancies. Both approaches present possible advantages and disadvantages, and these factors should be carefully considered to personalize and select the most appropriate genomic assay. This review focuses on the recent advancements of cfDNA-based genomic profiling assays in advanced solid tumors while highlighting the major challenges that should be tackled to formulate evidence-based guidelines in recommending the ‘right assay for the right patient at the right time’.

## 1. Introduction

**Figure 1:**
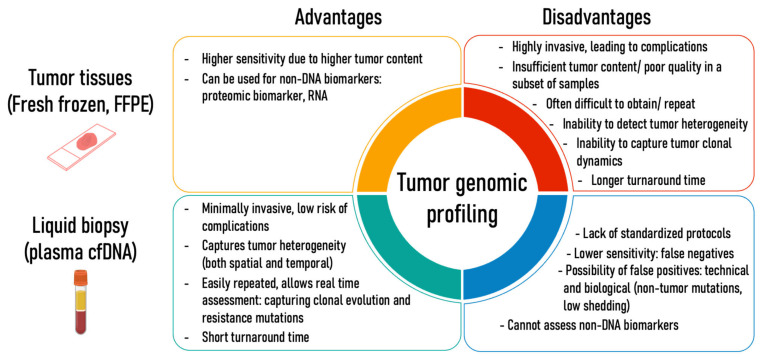
Advantages and disadvantages of tumor genomic profiling using tumor tissues and plasma cfDNA.

Technological advancements and reduction in sequencing costs have enabled genomic profiling of solid tumors to be performed routinely, which promoted the incorporation of precision oncology into the standard of care for advanced cancer patients [1]. Molecular profiling of tumor tissues, either from surgical resections or biopsy specimens, remains the standard approach to identify actionable genomic aberrations for molecular targeted therapies. However, the quality, quantity, and availability of tumor tissues from advanced cancer patients often pose challenges to the implementation of comprehensive genomic profiling (CGP) in clinical settings [2]. Recent multi-institutional studies have shown that 23–26% of collected tissue specimens could not proceed to CGP as a result of insufficient DNA quantity or tumor content [3,4]. Performing re-biopsy at recurrence is often difficult where the procedure is unfeasible in 20–60% of cases [5,6]. Furthermore, re-biopsy is also associated with potential complications and increased turnaround time, delaying treatment initiation [7,8,9]. Thus, the invasiveness of tissue biopsy may preclude re-biopsy at the time of recurrence and impede the identification of resistance mutations [10,11]. Furthermore, genomic profiling of tumor tissue provides a snapshot of a single point in space and time, lacking the ability to capture complex tumor heterogeneity and tumor clonal dynamics [12] (Figure 1).

Liquid biopsies, which involve genomic profiling of tumors using circulating biomarkers in the bodily fluid have emerged as a promising tool to complement and overcome the challenges of tissue-based CGP [13,14,15]. Among the different circulating biomarkers, circulating cell-free DNA (cfDNA) from blood has been most widely studied [16]. The origin and biology of cfDNA have been extensively discussed in previous reviews [17,18,19,20,21,22]. In brief, cfDNA is highly degraded DNA fragments released from apoptosis, necrosis, and secretion from cells [23,24]. The majority of cfDNA in plasma of healthy individuals originate from hematopoietic cells: 55% from white blood cells, 30% from erythroid progenitors, 10% from endothelial and 1% from hepatocytes [25,26,27]. In cancer patients, plasma cfDNA that originates from tumors, or commonly known as circulating tumor DNA (ctDNA), typically represents 0.01–90% of the total cfDNA found in blood [28]. Plasma ctDNA has been found to recapitulate the tumor’s molecular alterations, highlighting its potential to be used as a minimally invasive tumor marker in cancer patients [29]. Despite the low concentration of ctDNA present in blood, recent advancements in sequencing technology and bioinformatics enabled accurate detection of these ctDNA genomic alterations [30,31,32]. The accessibility and minimally invasiveness of blood sampling compared to tumor tissue biopsy allows genomic profiling to be conducted at multiple time points during cancer management, allowing real-time evaluation of treatment response and detecting clonal evolution during disease progression or recurrence [33,34,35]. Furthermore, a single blood sample may allow the capturing of ctDNA released from multiple tumor sites and regions, allowing the detection of inter- and intra-tumor heterogeneity that might be missed from a single-site tissue biopsy, depicting a more comprehensive and complete genomic profile of the tumor [36,37,38] (Figure 1).

The compilation of evidence that supports the use of ctDNA to identify actionable alterations from both analytical and clinical studies in recent years have advocated the FDA approval of several single-gene and multigene assays to be used as companion diagnostics matched to specific targeted therapies [30]. Despite the advantages of using liquid biopsy over tissue biopsy for CGP, limitations and challenges exist. cfDNA often presents at low concentrations (usually below 10 ng or 3000 genome copies per mL of plasma in cancer patients), and only a small fraction of cfDNA is tumor-derived [39]. Moreover, the tumor fraction of cfDNA varies between cancer types and even between metastatic patients with the same cancer type [40,41]. These variabilities and limited input material lead to the detection of ctDNA mutation being highly challenging, generating a higher false-negative rate in ctDNA analysis compared to tissue-based assays [17]. Similarly, false-positive findings as a result of biological factors such as non-tumor-derived clonal-hematopoiesis-related mutations present in plasma are a compelling issue that should be addressed to prevent misinterpretation of results [22,42,43,44]. The advantages and disadvantages of both plasma-based and tissue-based approaches stimulated an ongoing debate among researchers and clinicians as to whether a ‘plasma first’ or ‘tissue first’ approach is the most beneficial and appropriate genomic testing for advanced solid malignancies.

This review summarizes the recent advancements and supporting studies of the use of plasma ctDNA for genomic profiling in patients with advanced solid malignancies. In addition, we will also highlight the major challenges that should be tackled and factors that are to be considered to formulate evidence-based guidelines for the routine use of plasma-based genomic profiling in clinical settings.

## 2. Recent Advancements in ctDNA-Based Genomic Profiling Assays

Recent developments in sequencing technologies have increased the sensitivity of detecting the minute ctDNA present in plasma cfDNA with a higher level of accuracy and confidence (Table 1). Each method has its advantages and disadvantages. Polymerase chain reaction-based (PCR) methods such as BEAMing and droplet-digital PCR are fast, cost-effective, and simple to perform with extreme sensitivity and specificity of detecting mutations down to an allele frequency of 0.01% [28,45,46]. However, these target-specific approaches with limited multiplexing are only beneficial for the detection of a restricted number of known mutations, making them unsuitable for CGP of tumors. On the other hand, next-generation sequencing-based (NGS) approaches utilize multiplex PCR (amplicon-based) or hybridization capture to enrich and sequence the genomic regions of interest, enabling a more comprehensive analysis of the tumor genomic profile than the PCR-based methods. The larger NGS panel size also allows the evaluation of tumor mutation burden (TMB) and microsatellite instability (MSI), which are putative biomarkers for the response to immunotherapy [47,48]. However, due to the larger number of targets in NGS-based assays, the sensitivity of detection is often lower than the single-target approaches. Furthermore, errors acquired during NGS are one of the key contributing factors in limiting its accuracy and sensitivity to detect rare variants [49]. SafeSeq-S introduced the use of a unique identifier (UID) for error correction to increase the accuracy during sequencing. The UIDs are short sequences that are attached to each DNA template molecule that allows variant alleles present in the original sample to be distinguished from errors introduced during the template preparation and sequencing process [50]. The incorporation of UID has been shown to reduce the error rate by 70-fold [50]. The majority of ctDNA NGS assays available now incorporate unique identifiers, dual-indexing, and error suppression algorithms to increase the calling confidence of rare variants, thereby increasing their sensitivity and specificity [51,52]. These recent advancements, together with their supporting evidence, have encouraged the use of liquid biopsy for genomic profiling in clinical settings and received approval as in vitro companion diagnostics for molecular targeted therapies.

### 2.1. ctDNA Reflects the General Genomic Landscape of Tumors

Extensive work has been performed to compare the mutation detection concordance between tumor and ctDNA as an approach to evaluate the feasibility of utilizing ctDNA NGS-based assays for CGP of tumors in advanced cancer patients. The reported level of concordance by different studies has been greatly variable, ranging from 8.3% to 93% across different cancer types (Table 2). The differences in cohort size, study design, and definition of concordance may contribute to the variabilities observed. Despite these inconsistencies, the detection of oncogenic driver variants using NGS-based cfDNA assays has consistently demonstrated moderate to high sensitivity (75–93%) across studies in different cancer types [53,54,55,56,57,58,59]. In a large prospective, multicenter study that compared the detection of guideline-recommended biomarkers between a cfDNA-based and tissue-based assay in advanced and treatment-naïve non-small cell lung cancer (NSCLC), 80% of the genomic biomarkers detected from tumor tissues were concordantly detected from the plasma cfDNA [53]. The concordance level was observed to be 98.2% between tumor tissue and plasma for FDA-approved targets (*EGFR* exon 19 deletions and L858R, *ALK* fusions, *BRAF* V600E) [53]. A similar concordance level was observed for advanced breast cancer patients, where an overall concordance of up to 95% (negative and positive) was observed for the detection of mutations from the four major driver genes of breast cancer—*PIK3CA, ESR1, AKT1*, and *HER2* [54]. A high level of concordance was also reported for patients with advanced gastrointestinal (GI) cancer, where the most common treatment-relevant biomarkers in GI cancers—*KRAS*, *NRAS*, and *BRAF*—showed near 100% ctDNA sensitivity compared to tissue-based CGP [59]. In a recent study that evaluated the genomic landscape detected in ctDNA and tissue-based from 837 metastatic castration-resistant prostate cancer (mCRPC) patients, 75.3% of short variants were concordantly detected between the two assays [58]. More importantly, up to 89.7% of *BRCA1/2* alterations detected by tissue CGP were also detected from plasma cfDNA, highlighting the clinical utility of liquid biopsy CGP for the detection of clinically actionable alterations [58]. The high level of detection concordance for the major driver genes across different cancer types built the foundation for the use of ctDNA-based assay as an alternative to tissue-based CGP.

In addition to concordance analysis, several large cohort studies have been conducted in recent years to assess whether ctDNA NGS-based assays could benchmark against the current gold standard of tissue-based assays in detecting biomarkers for molecular targeted therapies. The detection of actionable alterations using ctDNA NGS-based assays has been consistent across studies, with approximately 40% of advanced cancer patients harboring at least one actionable target, comparable to tissue-based assays [59,60,61,62] (Table 2). In the NCI-MATCH study and the SHIVA study, actionable alterations were identified in approximately 40% of the evaluated patients using tissue-based CGP [60,62]. In one of the largest ctDNA studies conducted so far with over 10,000 advanced cancer patients, 41.2% of the patients were detected with at least one potential drug-sensitive target using a ctDNA-based CGP assay [40]. A comparable detection rate was observed in another pan-cancer study where 56% of recruited patients harbored clinically actionable alterations [63]. In the same study, the authors also demonstrated that the overall targetable alteration rate from ctDNA was similar to the patient-paired tissue [63]. Nakamura et al. also compared the identification of actionable alterations between two different GI advanced cancer cohorts that were either screened using ctDNA NGS (*n* = 1687) or using tumor tissues (*n* = 5621) [59]. The authors indicated that the detection rate was highly comparable in identifying targetable alterations from ctDNA and tissue (57.3% and 54.3% of patients, respectively) [59]. The variable actionable alteration detection rate was observed in different solid tumors (Table 2). Patients with advanced GI cancers showed the highest actionable alteration detection rate of 50%, followed by breast cancer and NSCLC at 38% and 36%, respectively (Table 2). In contrast, 30% of patients with prostate cancer harbor targetable alterations, and only less than 10% of thyroid and ovarian cancer patients were detected with actionable biomarkers using ctDNA-based CGP assays [40,58]. However, studies with larger cohort sizes are required to validate the detection rate of actionable alterations in rare cancer types. Several studies have also suggested that adding plasma NGS testing to the genomic profiling routine could increase targetable mutation detection by 48–75% and improve the delivery of targeted therapy in advanced cancer patients compared to the current standard approach [53,64].

### 2.2. Promising Clinical Outcomes following ctDNA Profiling for Treatment Selection

Recent clinical trials have incorporated exploratory objectives to evaluate the performance of ctDNA in identifying genomic markers for the prediction of treatment response for molecular targeted therapies. Several studies have shown patients with biomarkers of interest detected from cfDNA tend to exhibit a better prognosis than patients without the biomarker of interest, indicating their predictive value for treatment response [75,76,77,78,79]. In a prospective–retrospective study on archival plasma samples from the SoFEA and PALOMA3 trials, breast cancer patients with detected baseline *ESR1* mutations from plasma had improved progression-free survival (PFS) after being treated with fulvestrant (estrogen receptor antagonist) compared with exemestane (aromatase inhibitor), while patients with wildtype *ESR1* had similar PFS after receiving either treatment [75]. This was similarly observed in the SOLAR-1 study, where *PIK3CA*-mutated breast cancer, detected using plasma ctDNA, was associated with a better response to alpelisib plus fulvestrant than the fulvestrant arm [76]. The predictive value and clinical benefits of ctDNA genomic profiling have also been demonstrated in patients with carcinoma of unknown primary (CUP) [80]. CUP represents a heterogenous metastatic disease with an unidentifiable primary tumor where the standard treatments are often empiric chemotherapies with poor prognosis [81]. In the study conducted by Kato et al. 43% of the recruited 1931 CUP patients were detected with actionable alteration using plasma ctDNA CGP [80]. The authors also observed that patients treated with therapies with higher degrees of matching to ctDNA alterations showed better clinical outcomes [80].

The promising results from the exploratory studies suggested the potential of utilizing ctDNA CGP in clinical settings for genomic biomarker identification. The clinical benefits of ctDNA CGP were validated in recent large retrospective studies with cohort sizes of over 1000 patients. No significant differences were observed in the PFS and overall survival (OS) of patients selected based on ctDNA or tissues across several studies [59,66,68]. The clinical outcomes of liquid biopsy CGP compared to tissue CGP in advanced NSCLC patients were assessed in a multi-institutional, retrospective analysis of the real-world data [68]. The clinical and genomic data in this study were collected from a deidentified database where the majority of the patients were treated in a community setting. In this cohort of patients, a targetable genomic alteration was detected in 20% (188/937) of the cases that underwent ctDNA CGP compared to 22% (1215/5582) of tissue CGP cases. PFS for patients who received matched targeted therapy following liquid biopsy and tissue CGP were similar (13.8 vs. 10.6 months, respectively). Similarly, the overall response rate (partial/complete response) to matched targeted therapy was also comparable between post-liquid biopsy and post-tissue CGP (75% vs. 66%, respectively) [68].

In the past 2 years, several ongoing prospective phase II interventional clinical trials that were aimed to assess the accuracy and validity of ctDNA testing to select patients for genomic-directed therapies across different solid tumors have released their early results. All studies have shown over 99% of ctDNA sequencing success rates [54,82,83]. PlasmaMATCH is an open-label, multicohort trial of ctDNA testing in advanced breast cancer patients [54]. Recruited patients were subjected to ctDNA testing by NGS or droplet digital PCR and subsequently recruited into four parallel treatment cohorts matched to mutations (*AKT1*, *ESR1*, *HER2*, and *PTEN*) identified from plasma. A total of 34% of the sequenced patients had targetable mutations for cohort entry, and 13% of the patients entered one of the treatment arms. The *HER2* and *AKT1* arms reached the primary end point and exceeded the target number of responses where the response rate achieved by ctDNA-selection was comparable to that observed when guided by tissue testing [54]. However, the *ESR1* and *PTEN* arms did not reach the target number of responses, with only 8% and 11% response rates, respectively, similar to that previously reported [54]. A similar open-label, multicohort study was conducted for advanced NSCLC patients [82]. In the BFAST study, 2219 patients were screened using ctDNA-based NGS for detection of *ALK* rearrangements [82]. In total, 5.4% of tested patients were *ALK*-positive, and 3.9% of patients were enrolled and received the mutation-matched treatment alectinib. The *ALK*-positive cohort met its primary end point with an overall response rate of 87.4%, comparable to previous reports using tissue-based profiling [82]. These results confirmed the clinical application of ctDNA-based CGP as a method to detect genomic biomarkers for treatment selection in advanced solid malignancies, reaching comparable clinical outcomes to tissue-based profiling.

### 2.3. Shorter Turnaround Time (TAT) with Improved Clinical Trial Enrolment Rate

The overall high sequencing success rate and fast turnaround time (TAT) of ctDNA-based CGP are some of the key advantages of liquid biopsy over tissue profiling. Several studies have compared the TAT from sample collection to reporting results between the two CGP approaches. The median TAT for ctDNA-based NGS is 9 days (ranging 2–15 days) compared to 15 days for tissue CGP (ranging 12–20 days) [54,58,59,67,70,82,84,85]. The additional time required for scheduling tissue biopsy and the procedure itself may contribute to the longer TAT observed with tissue-based CGP compared to an in-clinic, same-day blood collection for plasma ctDNA analysis [86]. The significantly shorter TAT of cfDNA screening may allow earlier initiation of treatments, which can be particularly beneficial for aggressive and fast progression cancer types. Furthermore, the more rapid TAT may also indirectly increase trial enrollment rates compared to tissue-based assays without compromising the treatment efficacy. In the study that evaluated the clinical trial enrollment in advanced GI cancer, ctDNA profiling significantly shortens the screening duration from 33 days to 11 days when compared with using tumor tissues, and the trial enrollment rate was also improved by more than 5% [59]. It has been suggested that more patients in the tissue-profiling cohort would need to start an empirical therapy while waiting for the results, whereas more patients in the ctDNA genotyping cohort had results available in time to inform the selection of molecular targeted therapies, thereby increasing the overall clinical trial enrolment rate [59]. The expected TAT for the currently commercially available ctDNA CGP assays is within 7–10 days [87,88], which coincides with the current observations. However, the current TAT can potentially be further shortened with a more flexible and decentralized sequencing system, which can be placed at the point of care and operated with minimal technical supervision [89,90,91]. Such an automated NGS system would need further analytical and clinical validation for its use in clinical settings.

### 2.4. FDA Approval of Multigene ctDNA NGS Tests for CGP and as In Vitro Companion Diagnostics

In 2016, the U.S. Food and Drug Administration (FDA) approved the first ctDNA plasma-based genomic testing as a companion diagnostic for the detection of *EGFR* mutations to identify NSCLC patients that are eligible for erlotinib [92]. The Cobas EGFR Mutation Test v2 utilizes the RT-PCR technology, reaching a detection sensitivity of 0.1–0.8% [93] (Table 3) [93]. The approval of *EGFR* ctDNA testing provided a rapid and noninvasive method to detect clinically relevant genomic markers for treatment selection and has been proven to be reliable in clinical settings [94,95,96]. However, RT-PCR-based methods limit the number of testing targets and restrict their clinical applications. In 2020, the FDA approved two CGP liquid biopsy tests, Guardant360 CDx and FoundationOne Liquid CDx, for detecting genomic alterations from 55 and 311 genes, respectively (Table 3) [97,98]. Both panels were approved as complementary diagnostics for tumor mutation profiling in patients diagnosed with solid malignancy. The genomic findings from the ctDNA-CGP panels are to be used for treatment selection following professional guidelines [30]. Guardant360 CDx and FoundationOne Liquid CDx also received FDA approval as companion diagnostics for several molecular targeted therapies (Table 3) [99,100]. The number of companion diagnostic indications for both assays has increased since their initial approval and would likely continue to expand with the accumulation of evidence for other targeted therapies. The detection sensitivity for the approved targets using the Guardant360 CDx ranged from 0.2–0.5% [99], and 0.24–0.51% for the FoundationOne Liquid CDx [100]. In particular, to *EGFR* mutations, both approved ctDNA-CGP panels could not achieve the same level of sensitivity as the RT-PCR-based Cobas system, highlighting the difficulty to maintain the high sensitivity of mutation detection in large multiplexing systems.

## 3. Limitations and Challenges for the Routine Use of ctDNA-Based CGP for Treatment Selection

As we highlighted in Section 2.1 of this review, the reported level of concordance between tumor and plasma-based NGS analyses across studies has been greatly variable. Technical and biological factors which account for the generation of false-positive and false-negative results may contribute to the discordance observed. These factors remain the key limitations and challenges for the routine use of ctDNA profiling in clinical settings [17,101]. Furthermore, the lack of comprehensive guidelines to recommend the usage of ctDNA CGP also restricts its clinical use.

### 3.1. Technical Limitations Leading to False Positive and Negative Results

The major technical challenges that ctDNA NGS assays face are the small fragment size of cfDNA (~160 bps) and the low concentrations of ctDNA present in the blood. The detection of rare somatic mutations from such limited genomic material input is highly challenging [39]. Target enrichment, either by hybrid capture or amplicon methods, with extensive PCR amplification, is generally required to successfully capture the tumor genomic profile from the small quantity of cfDNA [102,103]. However, the small size of cfDNA fragments can restrict target enrichment and reduce the accuracy of alignment to the human reference genome [39,104]. These NGS chemical and physical factors can exacerbate biases and sequencing errors, resulting in both false-positive and false-negative results [105]. The majority of the current ctDNA NGS systems incorporate error-suppression strategies such as molecular barcodes or bioinformatic analyses. However, technical false positives remain [39,106]. In the study conducted by Stetson et al. the authors evaluated the false positive (FP) rates of four NGS gene panels using replicate sets of 24 plasma samples [106]. The positive predictive value ranged from 36 to 80% across the four vendors, and the majority of the FP variants occurred at less than variant allele frequency (VAF) of 1% [106]. The FP calls identified in the study were enriched for assay-specific mutational biases and mostly were novel variants not found in somatic variant databases [106]. In the study led by the Oncopanel Sequencing Working group, an average of 1.65–5.3 FP variants were observed per replicate across five leading ctDNA NGS assays [39]. The erroneous variants occurred almost exclusively at VAF less than 0.5% [39]. Despite the FP rate observed, the authors of this study concluded that the sensitivity, rather than precision, was the major determinant of discordance observed [39].

The current reported limit of detection by different ctDNA-targeted NGS platforms ranged from 0.004 to 2% [107]. However, the reproducibility and accuracy of alterations detected at low VAF have been variable across different platforms [39,106]. Similar to the FPs, the sensitivity of ctDNA assays drops significantly for mutations with low VAF [39,106]. Most of the commonly used ctDNA assays, both amplicon-based or hybrid capture, were highly sensitive for variants at high frequencies (over 90% sensitivity for VAF > 0.5%), but the sensitivity drops to 40% for alterations at VAF less than 0.5% [106]. This observation has been similarly reflected in clinical studies. In contrast to the high level of concordance observed in the major driver mutations, several studies have reported that the sensitivity of ctDNA mutation detection is dependent on their clonal fraction in the tumor tissue [42,59,63,108]. In an exploratory study conducted by Razavi et al. 72% of the genomic alterations detected from tumor biopsies were concordantly detected using an NGS-based ctDNA assay with no significant differences in the sensitivity across metastatic breast cancer, NSCLC, or castration-resistant prostate cancer [42]. However, the ctDNA detection rate was significantly higher for clonal mutations (75–90%) than subclonal mutations (20–30%) [42]. A similar disparity was observed in a large-scale study conducted on advanced GI cancer, where the positive predictive value was markedly higher for clonal alterations than for subclonal mutations (80.3 vs. 8.3%) [59]. Growing evidence indicates that subclonal mutations present in a small subset of tumor cells may confer resistance to targeted therapies [109,110,111,112]; therefore, improving the ability to identify these pre-existing resistance mutations from ctDNA is crucial to enhance the implementation of precision medicine. Recent studies suggest the enrichment of shorter fragment lengths, either using in vitro or in silico methods, may improve the detection of alterations with low VAF [113,114,115]. However, most of the studies were conducted with small sample sizes, and further studies are required to confirm the clinical implications of this approach.

The detection of targetable fusions or gene rearrangements from cfDNA remains technically challenging with inconsistent sensitivity across studies owing to the low prevalence of fusions in common solid tumors. To address this, Esagian et al. compiled 38 published studies that assessed the concordance of fusion detection between tumor tissues and plasma cfDNA in NSCLC patients [116]. A total of 1141 patients were included in the systematic review, and less than 60% of the samples with *ALK*, *RET*, and *ROS1* fusion from tumor tissues were concordantly detected from plasma cfDNA [116]. Most of the fusions arise from inter-chromosomal or intra-chromosomal conjunction of different introns, where the intronic regions can be extremely large with repetitive sequences (especially *ROS1* and *NTRK*) [117,118]. The inclusion of large intronic breakpoints as target regions may improve the detection of gene fusions; however, it may also increase the sequencing costs with reduced sequencing efficiency [117,118]. Current ctDNA-based CGP assays have focused on balancing the overall costs and the number of target probes to optimize the detection rate. FoundationOne Liquid assay included a selected number of introns for 9 of 16 targetable fusion kinase genes and was observed to have an overall concordance rate of 70% compared to tumor tissues from patients with various solid tumors [119]. Previous studies have also shown that assay optimizations such as the use of shorter amplicons or capture probes, primer extension, variant calling, and bioinformatic filtering may enhance the detection of fusions [120,121]. In contrast to cfDNA, cfRNA-based assays are not affected by intronic regions and only identify expressed fusion genes [117]. In a recent exploratory study, the authors demonstrated that a cfRNA-based NGS assay has an overall higher sensitivity to detect *ALK*, *ROS1*, and *RET* fusions than a cfDNA-based NGS system (78% and 33%, respectively) [122]. Furthermore, amplicon-based NGS panels that analyze gene fusions at the ctRNA level and gene alterations at the ctDNA level may also hold promise in improving fusion detection [89,123]. Larger studies are required to evaluate and confirm whether these strategies are beneficial in detecting fusions from plasma cell-free nucleic acids in the clinical setting.

### 3.2. Biological Limitations: Low Tumor Shedding and Non-Tumoral Origin of cfDNA

Besides the technical limitations, biological factors such as the location, size, and vascularity of the tumor may affect the release of ctDNA into the circulation, thereby compromising its detectability [124]. ctDNA fraction (the fraction of tumor-derived cfDNA) can vary significantly according to the tumor type and even between patients with the same tumor type (ranging from less than 1% to 80%) [40,42,125,126,127]. Tumors from the brain, renal, and thyroid have repeatedly been observed to have a lower ctDNA detection compared to colorectal, lung, and breast cancer, even at an advanced stage [40,125]. Furthermore, several studies have also reported that the detection rate of ctDNA is significantly higher in colorectal cancer patients with liver metastasis compared to nodal or lung metastasis [128,129,130]. The performance of ctDNA NGS assays was found to be highly dependent on the ctDNA fraction, particularly for detecting gene amplification [42,58,131]. For cfDNA samples with high tumor fractions (20–35%), 51–89% of copy number variations (CNVs) from tumor tissues were concordantly detected using ctDNA [58,132]. However, the sensitivity to detect CNVs from ctDNA drops to 28–35% for samples with low ctDNA fraction [58,132]. These biological factors should be taken into consideration when interpreting negative results from ctDNA CGP.

Non-tumoral variants detected from plasma contribute to the false-positive results detected from ctDNA-based CGP. Clonal hematopoiesis (CH) is a normal process of aging with the accumulation of somatic mutations in hematopoietic cells [22]. The detection of these non-tumor-derived CH mutations in plasma has been repeatedly reported as a source of biological noise to ctDNA genomic profiling [42,44,133,134,135]. Previous studies have shown that 15–53% of alterations detected from cfDNA of advanced cancer patients had features consistent with CH [42,43,133,136]. Moreover, a substantial number of CH variants detected from cfDNA are considered to be oncogenic and are indicated for targeted therapies, including mutations from *KRAS*, *EGFR*, and *PIK3CA* [42]. The VAF of CH mutations detected from plasma is indifferent to the tumor-derived mutations [44]. These features highlight the difficulties in distinguishing between CH and tumor-derived mutations and the risk of false findings. The significance of CH mutations in ctDNA CGP has been well recognized and acknowledged by researchers and clinicians. However, currently approved ctDNA-based CGP assays do not differentiate or report the origin of the mutations detected. This should be urgently addressed to prevent the initiation of inappropriate treatments as a result of false findings from ctDNA profiling.

### 3.3. Lack of Standardized Evidence-Based Guidelines for Tissue or Plasma First Approach

**Figure 2:**
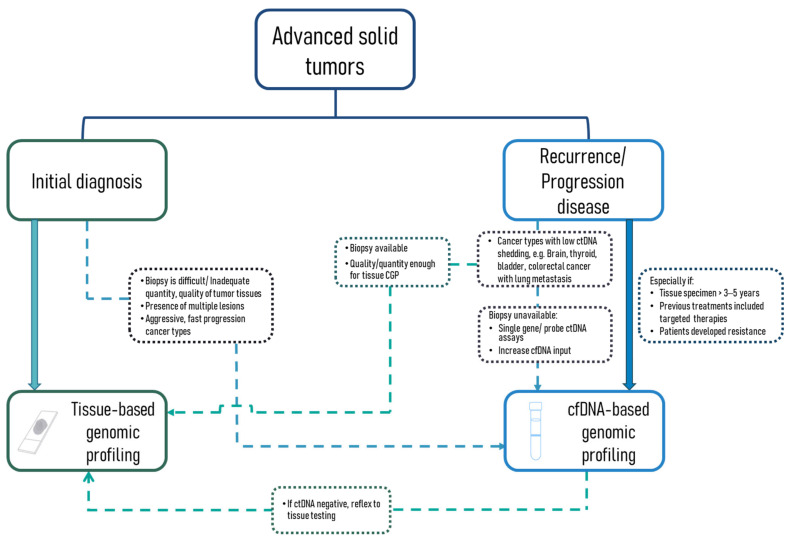
Generalized guidelines for CGP assay selection under different clinical situations. The schema was devised based on recommended guidelines from NCCN, ESMO, ASCO and IASLC together with existing literature. Patients with advanced solid tumors with known FDA-approved targeted therapies should undergo tissue-based genomic profiling for treatment selection. In cases where biopsy and tumor tissues are insufficient or unfeasible, patients present with multiple lesions or patients with aggressive, fast-progression cancers, cfDNA-based genomic profiling should be considered. CGP using cfDNA-based assays should be considered the assay of choice for patients who have developed recurrence or disease progression after targeted therapies. However, for patients with cancer types known to be low shedding of ctDNA and with contemporary tissues available, a tissue-based assay should be preferred. Patients with negative ctDNA results should reflex to tissue testing in all cases.

Due to the continuously growing evidence and recognition of the clinical use of ctDNA-based CGP for treatment selection, several leading professional organizations have included recommendations for the use of liquid biopsy in their clinical management guidelines. However, these recommendations were limited to NSCLC, breast cancer and prostate cancer patients. Furthermore, different multidisciplinary bodies also released contradicting suggestions as to whether a ‘plasma-first’ or a ‘tissue-first’ approach should be adopted in clinical settings. For the management of advanced breast cancer, both National Comprehensive Cancer Network (NCCN) and The European Society for Medical Oncology (ESMO) recommend ctDNA profiling as an alternative and complement to tissue CGP, while The American Society of Clinical Oncology (ASCO) recommends cfDNA as the specimen-of-choice for CGP [137,138,139]. Similarly, NCCN, ASCO and ESMO all recommend a ‘tissue-first’ approach during the initial diagnosis of NSCLC patients, while the International Association for the Study of Lung Cancer (IASLC) recommends the use of ctDNA CGP as the assay of choice in their latest consensus statement [140,141,142,143]. Both tissue-based and ctDNA-based CGP have their advantages and disadvantages, making it difficult to adopt a one-size-fits-all approach in the clinical setting. In this section, based on the accumulated evidence and recommendations from multidisciplinary expert panels, we have summarized some of the key factors that should be considered to select the most appropriate approach and proposed a generalized guideline for assay selection under different situations (Figure 2).

#### 3.3.1. Availability of Excision Tumor Tissue, Quality and Quantity of Biopsy; the Presence of Multiple Lesions; Cancer Types

Based on the observations from the current studies, the false-negative rate of plasma samples is higher than that of tissue-based assays [144]. In cases where surgical resection is performed and excision tumor tissues are available, tissue-based CGP would be preferred to overcome the lower sensitivity issue of ctDNA assays. This is also supported by the majority of the expert panels (NCCN, ESMO and ASCO), where ctDNA-based CGP is not recommended in the initial diagnosis setting [141,142,143]. However, other factors should also be considered to choose the most appropriate specimen for CGP. In cases where surgical resection is not feasible and only biopsy samples are available, the timing and condition of the patient are crucial for assay selection. In cases where biopsy samples are difficult to retrieve and the quality and quantity of tissue specimens are insufficient for tissue-based CGP, liquid biopsy should be considered the assay of choice. Furthermore, if patients present with multiple lesions or metastases, liquid biopsy should be preferred due to its ability to detect intertumoral heterogeneity, which could be missed by a single tissue testing [145,146,147]. The detection of spatial tumor heterogeneity was highlighted in a study that sequenced paired primary tumor, metastatic tissue, and plasma cfDNA of breast cancer patients [146]. Plasma cfDNA detected up to 97% of alterations from primary and metastatic tissues, and 13 of the variants in metastatic tumors were exclusively detected from ctDNA, and not in the corresponding primary tumors [146]. However, currently approved ctDNA CGP assays would not be able to identify the origin of the mutations (inter- or intratumoral heterogeneity) without previous knowledge of the tumor genomic profile. Future studies exploring the use of methylation and fragmentomic features of cfDNA may help identify their cellular origins [148,149]. The shorter TAT of cfDNA-based CGP also suggests that liquid biopsy would be more beneficial than tissue profiling for aggressive and fast progression cancers, allowing earlier treatment commencement [144].

#### 3.3.2. Timing: Initial Diagnosis or Recurrence/Progression Disease

At initial diagnosis, tissue-based CGP assays are likely to be more beneficial than ctDNA profiling for treatment-naïve advanced cancer patients with resectable tumors, owing to the lower sensitivity of ctDNA-based assays. However, for treatment selection at the time of recurrence or during disease progression, ctDNA-based genomic profiling should be preferred [144]. Several studies have shown that the longer collection interval between plasma and tissues leads to higher discordance [63,65,70,130,150,151]. The most striking area of discordance between liquid and tissue CGP is often the detection of a range of resistance mutations from liquid biopsy [58,152,153]. In a study that evaluated the detection of androgen receptor (AR)-activating alterations in prostate cancer, for samples that were collected more than 30 days apart and who had previous exposure to AR signaling inhibitors during the collection interval, only 5% of the AR short variants detected from plasma were concordantly detected from tumor tissues. This highlights the ability of liquid biopsy to detect resistance variants that may not be detected from archival tumor tissues and could provide additional ability to identify patients who might benefit from a non-AR signaling inhibitor [58]. Similarly, an increased discordance in the drivers of resistance to anti-EGFR therapy: *KRAS*, *NRAS*, and *EGFR* mutations were observed in metastatic colorectal cancer patients who were treated with anti-EGFR therapy than the treatment-naïve patients (concordance rate of 71% and 94%, respectively) [153]. The clinical benefits of the ctDNA-based assay over tissue-based CGP in detecting resistance mutations at the point of disease progression are also recognized by NCCN, ESMO, and IASLC, where initial use of ctDNA testing for *EGFR*-T790M alterations is preferred in patients that have developed progression from EGFR tyrosine kinase inhibitors (TKIs) [140,141,143]. The ability of ctDNA profiling to capture temporal tumor heterogeneity highlights the advantage of liquid biopsy over tissue biopsy in patients who have developed recurrence or received previous therapies. However, for cancer types with known low ctDNA tumor fraction and poor ctDNA detection sensitivity (e.g., brain, renal, thyroid, and colorectal cancer with lung metastasis), conventional tissue-based CGP or single-gene cfDNA assays with higher assay sensitivity should be considered instead [144].

## 4. Future Perspectives of ctDNA-Based CGP to Maximize Its Utilities in Personalizing Oncology Management

The use of liquid biopsy is increasingly being incorporated into the clinical protocol for targeted treatment guidance; however, there are still several areas that require further research. Here, we provide our perspectives on the key challenges that should be attended to optimize the use of ctDNA-based CGP in oncology management.

### 4.1. Standardizing Methods to Exclude CH Mutations

One of the main challenges for the clinical use of ctDNA-based CGP is the lack of standardized methods to determine the origin of the alterations detected from plasma and the exclusion of CH-related mutations. Most of the studies conducted so far utilize paired-sequencing of the matched white blood cells to a comparable depth as cfDNA to filter out CH mutations [42,44,154]. This approach remains useful; however, it incurs additional costs, which hampers its practicality in clinical settings. Alterations detected from cfDNA may also be validated using white blood cells with single-gene assays such as droplet digital PCR. Single probe assays often have higher sensitivity with lower running costs than NGS; however, additional validation assays would prolong TAT and delay the initiation of treatments. In contrast to validating using white blood cells, recent studies have focused on utilizing cfDNA fragmentomic analysis to differentiate and determine the origin of cfDNA mutations [22,134,135,155,156]. Several studies have shown that ctDNA presents as shorter fragments than CH or non-mutated cfDNA fragments, which might be useful for distinguishing the tumor-derived mutations [63,135,155,156]. More importantly, fragment size distribution can be determined without additional sequencing or validation assays, thereby minimizing costs and time, making it ideal for clinical implementations. However, current observations are based on proof-of-concept studies with small sample sizes. Larger studies are required to confirm the clinical validity. Besides the fragment size of cfDNA, the fragmentation pattern, which includes the nucleotide motifs at the fragment ends, single-stranded jagged ends, and the genomic locations of the fragmentation endpoints, has been suggested to relate to the tissue of origin [149]. It is currently unclear whether these unique characteristic signatures can be employed to distinguish tumor-derived mutations. Future studies should evaluate and determine the most appropriate and economical method to exclude non-tumor-derived alterations.

### 4.2. Establishing the VAF Threshold for Treatment Initiation

Current technological developments have been mainly focusing on improving the limit of detection to improve the ctDNA detection sensitivity. However, limited research has been conducted to evaluate the clinical outcomes of targeting alterations that are detected at low VAF using ctDNA profiling. There are no cutoff values or thresholds from the guidelines of the approved companion diagnostics to help guide clinicians on whether treatments should be initiated based on the reported VAF. It is unclear whether targeting alterations detected at low VAF from plasma could result in clinical benefits. Two recent exploratory studies have observed no significant differences in treatment response between NSCLC patients detected with *EGFR* mutations below or above ctDNA VAF of 1% [66,67]. The authors from both studies also reported that a trend of greater clinical benefit was observed in those with a low VAF (<1%), suggesting better disease control in those patients with lower tumor burden as reflected by ctDNA [66,67]. On the other hand, *EGFR* clonal dominance determined by plasma cfDNA was observed to be independently associated with improved efficacy of EGFR-TKIs in patients with advanced NSCLC [157]. In the study conducted by Ai et al. the authors employed a hierarchical Bayesian clustering method to analyze the clonal structure in ctDNA and evaluated whether the actionable *EGFR* mutation was the dominant clone across 300 treatment-naïve advanced NSCLC patients [157]. The objective response rate and PFS were significantly higher for patients with *EGFR* as a dominant clone than those nondominant clones, according to plasma ctDNA NGS results [157]. The authors suggested that the ctDNA VAF normalized using a statistical model might be a more stable parameter for guiding therapeutic strategies based on ctDNA results [157].

### 4.3. Frequency of ctDNA CGP for Treatment Optimization

A series of observational and interventional clinical trials have demonstrated that monitoring of clonal dynamics and the development of resistance mutations using serial ctDNA analysis may assist in treatment optimization. However, the frequency of ctDNA monitoring and optimal sampling timepoints to achieve maximal clinical benefit remains unclear. Previous studies have shown that ctDNA CGP before the initialization of re-challenge therapy could be effective in predicting clinical benefit [158,159]. In a phase II study, patients with wild-type *RAS/BRAF* ctDNA before initialization of anti-EGFR re-challenge have a significantly longer OS compared to patients with mutated ctDNA (17.3 and 10.4 months, respectively) [158]. Similarly, in a recent single-arm interventional clinical trial, metastatic colorectal patients who have developed resistance to anti-EGFR monoclonal antibodies were screened for wild-type *RAS/BRAF/EGFR* using ctDNA for selection of re-challenge therapy [159]. The primary endpoint of the clinical trial was met with an overall disease control rate of 59%, corroborating the effectiveness of ctDNA CGP in selecting patients for re-challenge therapy [159]. Furthermore, Parseghian et al. have demonstrated that ctDNA evaluation at 4.4 months after the cessation of anti-EGFR therapy may be the optimal timing to assess the regression of resistant *RAS/BRAF/EGFR* clones and to guide the initiation of anti-EGFR re-challenge therapy for maximal clinical benefits [160].

Early detection of resistance mutations through serial ctDNA analysis during treatment has been suggested as an indicator for treatment intervention to prevent or delay tumor progression [161,162,163,164]. The ability of ctDNA to detect the emergence of resistance mutations and prediction of recurrence has been well reported; however, the frequency of ctDNA monitoring has been variable across studies, ranging between fortnightly to every 3 months [161,162,163,164]. The feasibility of preventing or delaying tumor progression via ctDNA monitoring was first evaluated in the recent phase III PADA-1 trial. Metastatic breast cancer patients receiving first-line treatment with palbociclib plus aromatase inhibitor therapy were monitored using ctDNA every 2 months and were switched from an aromatase inhibitor to fulvestrant as soon as an *ESR1* mutation became detectable from ctDNA [163]. The early results from the trial indicated that patients who switched to fulvestrant co-treatment showed a 39% reduction in the risk of disease progression or death with a PFS of 11.9 months compared with 5.7 months in patients that maintained the aromatase inhibitor co-treatment [163]. The results from the trial highlighted the clinical benefits of ctDNA monitoring for early detection of resistance mutations to personalize and modify treatment regimens.

The use of a large cfDNA panel or a targeted approach to longitudinally monitor patients with advanced-stage disease should also be further investigated. It has been suggested that large ctDNA CGP panels may be more beneficial in cases where resistance mechanisms of the drugs are not known, while in settings where resistance mechanisms are well described, longitudinal ctDNA monitoring using a targeted approach may be more appropriate and cost-effective [30]. Large studies with health economic benefit assessments are required to facilitate the smooth translation of ctDNA monitoring into the clinical setting.

### 4.4. ctDNA Biomarkers for Immunotherapy

Tumor mutation burden (TMB) and microsatellite instability (MSI) have shown to be effective genomic biomarkers in identifying patients who are likely to benefit from immune checkpoint inhibitors [165]. However, the insufficiency and poor quality of tissue sampling prevented TMB and MSI testing from being performed regularly in the current clinical setting [166]. ctDNA CGP using large NGS panels may overcome these shortcomings and may serve as a detection tool for prognostic and predictive biomarkers for immunotherapy [47,48]. Modest but consistent level of correlation (mean of R = 0.6) between TMB determined from tissues (tTMB) or ctDNA (bTMB) has been reported across different studies [47,167,168,169,170]. Tumor heterogeneity and low ctDNA tumor fraction from blood may account for the absence of a higher level of concordance between tTMB and bTMB [47,170,171]. Nevertheless, similar to tTMB, bTMB was found to be predictive of immunotherapy outcomes [170,172]. In a meta-analysis study that evaluated the results from 6 randomized clinical trials with a total of 2338 advanced NSCLC patients who were treated with PD-1/PD-L1 inhibitors, patients with high bTMB showed significantly better OS, PFS, and objective response rates from immunotherapy than patients with low bTMB [172]. In contrast to bTMB, ctDNA CGP has shown high sensitivity (78–87%) in detecting MSI compared to tumor tissues [173,174,175]. Patients detected with MSI using ctDNA assays also demonstrated significantly prolonged PFS, confirming their potential clinical validity [173,174,175]. The accumulating observations should be validated in large cohort studies, and future studies should emphasize the standardization of bTMB and MSI assays and determine a validated threshold to accelerate their translation to the clinics.

## 5. Conclusions

Research developments and the accumulation of analytical and clinical evidence for the use of ctDNA genomic profiling from the past decade have transformed our clinical practice in oncology. The approval of ctDNA-based assays for CGP and as companion diagnostic tools have allowed more cancer patients to gain access to targeted therapies and supported the realization of precision oncology. Developing evidence-based guidelines for the use of ctDNA profiling and addressing the current limitations, such as the exclusion of CH alterations, will further optimize the clinical usage of liquid biopsy for treatment selection. Future studies should focus on expanding the current roles of plasma ctDNA to improve patient access to precision medicine and thereby improve patient outcomes.

## Figures and Tables

**Table 1:** Common ctDNA detection platforms.

Method	Name	Example	Number of Targets	LOD
PCR-based	qPCR	COLD-PCR	1	0.1–1%
	dPCR	BEAMing	1–20 targets	0.01–0.1%
	dPCR	ddPCR	up to 5 targets	0.01–0.1%
	MassSpec PCR	UltraSeek	Multigenes	0.1–1%
NGS-based	Amplicon-based	IonTorrent-Oncomine	Multigenes	0.1%
		Safe-SeqS; Plasma-SeqSensei	Multigenes	0.04–0.2%
	Hybrid capture	Avenio, TruSight 500	Multigenes	0.5%
		CAPP-Seq; Guardant360; FoundationOne Liquid	Multigenes	0.02%

PCR: Polymerase Chain Reaction; ddPCR: droplet digital PCR; qPCR: quantitative PCR; NGS: Next-generation sequencing; COLD-PCR: Co-amplification at lower denaturation temperature PCR; BEAMing: Beads, emulsion, amplification, magnetics; LOD: Limit of detection.

**Table 2:** Summary of recently published studies on the mutation detection concordance between tumor tissues and ctDNA.

Cancer Type	Sample Size	Method	Number of Genes	Types of Variants	Detection Rate	Detection of Actionable Mutations from ctDNA	CH Elimination	Tissue Plasma Concordance *	Comments	Reference
Pan Cancer	11,525	Customized hybridization capture NGS	1021	SNVs, Indels, CNVs, Fusion, bTMB	73.50%	41.2%	Yes	N.D		[40]
Pan Cancer	681	MSK-ACCESS hybridization capture NGS	129	SNVs, Indels, CNVs, Fusion, bTMB	73%	56.0%	Yes	59.0%	Variable collection interval between tissue and plasma	[63]
Pan Cancer	433	Guardant 360 hybridization capture NGS	73	SNVs, Indels, CNVs, Fusion	37%	N.D.	No	45.0%	Only examined TP53; variable collection interval between tissue and plasma	[65]
Pan Cancer	161	Customized hybridization capture NGS (GRAIL)	508	SNVs, Indels, CNVs, Fusion	84%	N.D.	Yes	72.0%		[42]
Pan Cancer	10,593	Guardant 360 hybridization capture NGS	73	SNVs, Indels, CNVs, Fusion	86%	72.0%	No	92.0%	Concordance based on 543 patients, in 7 genes	[57]
Lung	1971	Guardant 360 hybridization capture NGS	73	SNVs, Indels, CNVs, Fusion	87.30%	26.70%	No	N.D.		[66]
Lung	262	Guardant 360 hybridization capture NGS	73	SNVs, Indels, CNVs, Fusion	60.40%	60.40%	No	67.70%	Initial diagnosis/treatment naïve; only examined 6 genes (*EGFR, ALK, MET, ROS1, RET, KRAS*)	[67]
Lung	934	FoundationLiquid/FoundationACT NGS	62/70	SNVs, Indels, CNVs, Fusion	90.00%	20.00%	No	N.D.	ctDNA: 937 patients; Tissue: 5582 patients	[68]
Lung	8388	Guardant 360 hybridization capture NGS	73	SNVs, Indels, CNVs, Fusion	86.00%	48.00%	No	N.D.		[69]
Lung	282	Guardant 360 hybridization capture NGS/ddPCR	73	SNVs, Indels, CNVs, Fusion	-	27.30%	No	80.0%		[53]
Lung	127	Customized hybridization capture NGS (GRAIL)	37	SNVs, Indels, CNVs, Fusion	-	-	Yes	75.0%		[56]
Lung	210	ResBio ctDx-Lung amplicon-based NGS	21	SNVs, Indels, CNVs, Fusion	64.30%	21.90%	No	60.6%	A subset of patients subjected to treatment at the time of plasma collection	[70]
Lung	323	Guardant 360 hybridization capture NGS	73	SNVs, Indels, CNVs, Fusion	-	33.00%	No	N.D.		[64]
Breast	162	Customized amplicon-based NGS	39	SNVs	92.50%	39.00%	No	N.D.		[71]
Breast	1044	Guardant 360 hybridization capture NGS/ddPCR	ddPCR: 4; NGS: 73	SNVs, Indels, CNVs, Fusion	51.10%	34.50%	No	93%	Concordance is based on 77 patients in 4 genes (*AKT1, HER2, ESR1, PIK3CA*). Negative concordance was included.	[54]
Breast	255	Guardant 360 hybridization capture NGS	73	SNVs, Indels, CNVs, Fusion	89.00%	26.00%	No	79–91%	Actionable alterations in *PIK3CA, ESR1, ERBB2*	[55]
Gastrointestinal	1687	Guardant 360 hybridization capture NGS	73	SNVs, Indels, CNVs, Fusion	91.40%	57.30%	No	8.3–80.3% (<0.3 vs. >0.3 clonality)	Concordance is based on 287 patients	[59]
Gastrointestinal	200	Customized amplicon-based NGS	150	SNVs, Indels, CNVs, Fusion, bTMB, bMSI	84.05%	45.50%	No	N.D.		[72]
Gastrointestinal	1064	Guardant 360 hybridization capture NGS	73	SNVs, Indels, CNVs, Fusion	93.70%	47.70%	No	N.D.	Only included metastatic colorectal cancer patients	[73]
Gastrointestinal	282	Guardant 360 hybridization capture NGS	73	SNVs, Indels, CNVs, Fusion	75.00%	48.00%	No	50–86%		[74]
Prostate	3334	FoundationLiquid/FoundationACT NGS	62/70	SNVs, Indels, CNVs, Fusion	79.50%	30% (DDR gene alteration)	Yes	75.3% (SNVs); 70.3% (rearrangements); 27.5% (CNVs)	DDR alterations: *BRCA1/2; CDK12*; MSI-H	[58]

* % of mutations detected from tumor tissues also detected from plasma cfDNA, unless stated. CH: clonal hematopoiesis; ddPCR: droplet digital PCR; N.D.: not determined; SNVs: single nucleotide variants; CNVs: copy number variations; bTMB: blood tumor mutation burden; DDR: DNA damage response and repair; MSI-H: microsatellite instability high.

**Table 3:** Summary of FDA-approved diagnostic plasma ctDNA assays.

Approved Diagnostic Tool	Technology	Number of Genes	Input (ng)	Disease	Drug	Biomarker	LOD
Cobas EGFR	RT-PCR	1	Undefined; (2 mL of plasma)	NSCLC	Erlotinib & Gefitinib	*EGFR* Exon 19 deletions; L858R	Exon 19 deletions: 0.1–0.5%; L858R: 0.4–0.8%
					Osimertinib	*EGFR* T790M	Exon 19 deletions: 0.1–0.5%; L858R: 0.4–0.8%; T790M: 0.4–0.8%
Therascreen	RQT-PCR	1	Undefined; (2 mL of plasma)	Breast	Alpelisib	*PIK3CA* (C420R, E542K, E545A, E545D [1635G > T only], E545G, E545K, Q546E, Q546R; and H1047L, H1047R, and H1047Y)	1.82–7.07%
FoundationOne Liquid CDx	NGS-hybridization enrichment	324 (311 FDA approved)	20	NSCLC	Alectinib	*ALK* rearrangements: *ALK*-*EML4*	*ALK*-*EML4*: 0.24%
					Osimertinib & Erlotinib	*EGFR* Exon 19 deletions; L858R	Exon 19 deletions: 0.27%; L858R: 0.34%
					Capmatinib	*MET* SNVs & Indels that lead to *MET* exon 14 skipping	Substitutions: 0.4%; Indels: 0.28%
				Prostate	Olaparib	*BRAC1*	Substitutions: 0.34%; Indels: 0.38%
						*BRCA2*	Substitutions: 0.37%; Indels: 0.36%;
						*ATM* alterations	Indels: 0.51%
					Rucaparib	*BRCA1*	Substitutions: 0.34%; Indels: 0.38%
						*BRCA2*	Substitutions: 0.37%; Indels: 0.36%
				Ovarian	Rucaparib	*BRCA1*	Substitutions: 0.34%; Indels: 0.38%
						*BRCA2*	Substitutions: 0.37%; Indels: 0.36%
				Breast	Alpelisib	*PIK3CA* (C420R, E542K, E545A, E545D [1635G > T only], E545G, E545K, Q546E, Q546R; and H1047L, H1047R, and H1047Y)	Substitutions: 0.34%
Guardant360 CDx	NGS- hybridization enrichment	74 (55 FDA approved)	30	NSCLC	Osimertinib	*EGFR* Exon 19 deletions; L858R; T790M	0.20%
					Amivantamab-vmjw	*EGFR* exon 20 insertions	0.30%
					Sotorasib	*KRAS* G12C	0.50%
